# Curcumin Relaxes Precontracted Guinea Pig Gallbladder Strips via Multiple Signaling Pathways

**DOI:** 10.14740/gr689w

**Published:** 2015-10-21

**Authors:** Loren W. Kline, Edward Karpinski

**Affiliations:** a School of Dentistry, University of Alberta, Edmonton, Alberta T6G 2E1, Canada; b Department of Physiology, University of Alberta, Edmonton, Alberta T6G 2H7, Canada

**Keywords:** Curcumin, Calcium channels, Potassium channels, Gallbladder, Smooth muscle

## Abstract

**Background:**

Curcumin (diferuloymethane) is the active ingredient of the dietary spice turmeric. Curcumin modulates various signalling molecules, including inflammatory agents, transcription factors, protein kinases and cell cycle regulatory proteins. The purpose of this study was to determine if curcumin had an effect on gallbladder motility.

**Methods:**

A pharmacologic *in vitro* technique was used. Since curcumin relaxed both cholecystokinin octapeptide- (CCK) and KCl-induced tension of guinea pig gallbladder strips in a concentration dependent manner, an *in vitro* technique was used to determine which second messenger system(s) mediated the observed relaxation. Paired *t*-tests, *t*-tests and analysis of variance were used for statistical analysis. Differences between mean values of P < 0.05 were considered significant.

**Results:**

To determine if protein kinase A (PKA) mediated the curcumin-induced relaxation, PKA inhibitor 14-22 amide myristolated (PKA-IM) was used. PKA-IM had no significant effect on the amount of curcumin-induced relaxation. When the protein kinase C (PKC) inhibitors bisindolymaleimide IV and chelerythrine Cl^-^ were used together, a significant (P < 0.01) reduction in the curcumin-induced relaxation was observed. The use of tetraethylammonium chloride (TEA) caused a significant (P < 0.01) decrease in the amount of curcumin-induced relaxation. Adding curcumin prior to the KCl caused a significant (P < 0.001) decrease in the amount of KCl-induced tension.

**Conclusions:**

The results suggested that the curcumin-induced relaxation is mediated by multiple signaling pathways including the PKC second messenger system, inhibiting extracellular Ca^2+^ entry and K+ channels.

## Introduction

Curcumin (diferuloylmethane) is a polyphenolic compound isolated from the rhizomes of the medicinal plant *Curcuma longa* (turmeric). It is the active ingredient of the dietary spice turmeric. Turmeric has been used for medicinal purposes for thousands of years [1]. Curcumin has many beneficial effects including antioxidant, antiviral, antifungal, antibacterial, anti-inflammatory, and anti-cancer activities [1, 2]. Curcumin has been shown to have cardiovascular protective effects. It decreased the development of heart failure and atherosclerosis [3, 4]. Curcumin had an inhibitory effect on voltage-dependent K^+^ channels in rabbit coronary arterial smooth muscle cells [5]. Curcumin significantly reduced the activity of protein tyrosine kinase in rat aortic vascular smooth muscle cells and decreased the development of heart failure [3, 6-8].

Curcumin increased contractility of the rat urinary bladder, and caused a concentration-dependent increase of muscle tone in urinary bladders isolated from Wistar rats. The increase in muscle tone was mediated by the curcumin activation of the muscarinic M-1 cholinoceptors (M_1_-mAChR) which then increased muscle tone through the phospholipase C-protein kinase C (PLC-PKC) pathway [9]. While curcumin increased contractility in rat urinary bladders, it decreased gastric emptying in albino rats [10]. Curcumin suppressed the proliferation of and induced apoptosis of biliary cancer cells through the modulation of multiple signaling pathways [11].

Studies were reported to determine if curcumin was a suitable cholecystokinetic agent for preventing gallstones in patient with a high risk, e.g., those in long standing fasting periods, sepsis or receiving complete parenteral nutrition [12].

The purpose of this study was to determine if curcumin had a cholecystokinetic effect, and if not, did curcumin relax either cholecystokinin- or KCl-induced tension. Since curcumin caused relaxation of both cholecystokinin octapeptide- (CCK) and KCl-induced tension, the study focussed on determining the mechanism which mediated the relaxation.

## Materials and Methods

The experiments were performed under a protocol (#275) re-approved (February 3, 2015) by the Animal Care Committee-Health Sciences of the University of Alberta. Male Hartley guinea pigs (200 - 350 g body weight) were killed by decapitation. The gallbladder was removed from the guinea pig, fat and connective tissue were removed from the gallbladder, and the gallbladder was placed in Krebs-Henseleit solution (KHS) that was gassed with 95% O_2_ and 5% CO_2_. The composition of the KHS was (in mM) NaCl, 115; KCl, 5; CaCl_2_, 2.1; MgSO_4_, 1.2; NaH_2_PO_4_, 1.2; NaHCO_3_, 25; and glucose, 11. Each gallbladder was cut into strips (1.5 × 0.5 cm) and maintained in Sawyer-Bartlestone chambers filled with KHS, maintained at 37 °C, and gassed with 95% O_2_ and 5% CO_2_. An optimum resting tension of 0.7 g was determined previously and used in the study [13-15].

The force developed by the gallbladder strips was measured with Grass FT03 force displacement transducers (Grass Instruments Co., Quincy MA, USA) and recorded on a Grass 7D polygraph (Grass Instruments Co., Quincy, MA, USA). Isolated strips were equilibrated in the chambers for 45 min prior to determining their suitability for use. Each chamber had 2 μM (final concentration) atropine added, in every experiment, 3 min prior to the addition of 1.0 nM CCK. The tension was measured. This was followed by three changes of KHS. The test was repeated twice with 25 min between tests. A repeatable minimum active tension of 0.5 g had to be generated by the strips before use. All agents used were added directly to the chambers. All concentrations are reported as the final concentration in the chambers.

Several series of experiments were performed to examine the effects of curcumin on tension generated by the gallbladder strips. Initially curcumin was added to the chambers to determine if it would stimulate contraction of the strips. Concentrations of 25, 50, and 100 mM were used. No tension was developed after adding curcumin to the chambers.

CCK (1 nM) was found to produce a stable long lasting tension after 3 min. This steady tension lasted at least 10 min [13, 16]. In order to determine if curcumin could relax CCK- or KCl-induced tension, concentration response curves were generated. The CCK-induced tension was allowed to reach a steady level (3 min). The strips were exposed to a concentration of curcumin, the response was observed until the relaxation reached a steady level (approximately 5 min), the KHS was changed three times, and the strips were allowed to recover for 30 min, before testing a different concentration of curcumin. The concentration of curcumin (50 mM) was selected for use in subsequent experiments as it produced a reproducible relaxation. The same procedure was followed to generate a concentration response curve using 40 mM KCl instead of 1 nM CCK. KCl directly depolarizes smooth muscle, and its use is a standard procedure.

In order to determine if the Ca^2+^ released from the endoplasmic reticulum mediated the curcumin-induced relaxation 2-aminoethoxydiphenylborane (2-APB) (125 μM), a cell permeable inhibitor of IP_3_-induced Ca^2+^ release, was added to the chambers 10 min prior to the CCK. The CCK was then added to the chambers. When the tension reached a steady level, 50 mM curcumin was added to the chambers. The amount of relaxation was observed. The amount of relaxation observed when curcumin only was used was then compared to the amount of relaxation observed when the strips were treated with 2-APB and curcumin. This procedure was followed with each agent used.

When the protein kinase A (PKA) inhibitor PKA inhibitor 14-22 amide myristolated (PKA-IM; 180 nM) was used, it was added to the chambers 15 min prior to CCK to ensure adequate time for entry into the smooth muscle. The use of KT5823 (585 nM), a PKG blocker, was added to the chambers 5 min prior to the addition of CCK. Genistein (10 μM), a protein tyrosine kinase inhibitor, was added to the chambers 5 min prior to the addition of CCK.

The protein kinase C (PKC) inhibitors, chelerythrine Cl^-^ (5 μM) and bisindolymaleimide IV (BIM, 0.5 μM), were used together to determine the effects of PKC on curcumin-induced relaxation of CCK-induced tension. BIM blocks the translocation of PKC to its site of action. Chelerythrine Cl^-^ acts on the catalytic domain of PKC. It was shown previously that using BIM and chelerythrine Cl^-^ in combination produced the most consistent results [13, 16]. They were added to the chambers 5 min prior to the CCK.

L-N^G^-methyl-L-arginine acetate salt (L-NMMA; 20 μM), a nitric oxide (NO) synthase inhibitor, was used to determine if NO mediated the curcumin-induced relaxation.

In order to determine if curcumin inhibited extracellular Ca^2+^ entry, 40 mM KCl was used to induce tension in the strips. After the amount of tension generated by the 40 mM KCl was recorded, the KHS was changed three times, and the strips allowed equilibrating for 25 min. Fifty mM curcumin was then added to the chambers 3 min prior to the addition of 40 mM KCl. The amount of tension generated was recorded and compared to that observed when the KCl was added to the chambers with no curcumin.

Tetraethylammonium chloride (TEA, 5 μM) was used to determine if the effects of curcumin were mediated by inhibiting K^+^ channels. TEA was added to the chambers 3 min prior to the CCK.

The following agents were purchased from Sigma Chemical Company (St. Louis, MO, USA): CCK, atropine, L-NMMA, TEA and bisindolymaleimide IV. The following agents were purchased from EMD Millipore (Etobicoke, Ontario, Canada) PKA-IM, KT5823, chelerythrine Cl^-^ , genistein and 2-APB. Curcumin was purchased from Cayman Chemicals (Ann Arbor, MI, USA). All agents were dissolved in either distilled water or dimethyl sulfoxide (DMSO). The amount of DMSO (10 μL) added to the chambers was determined to have no effect on the strips.

### Statistical analysis

Statistical comparisons were done using either the *t*-test, paired *t*-test or analysis of variance. Results are expressed as mean ± SE. Differences among mean values with P < 0.05 were considered significant. The number of gallbladders (animals) used in each experiment are indicated by “n”. Each gallbladder was used to prepare four strips; therefore, an “n” of 4 represents the use of up to 16 strips.

## Results

**Figure 1:**
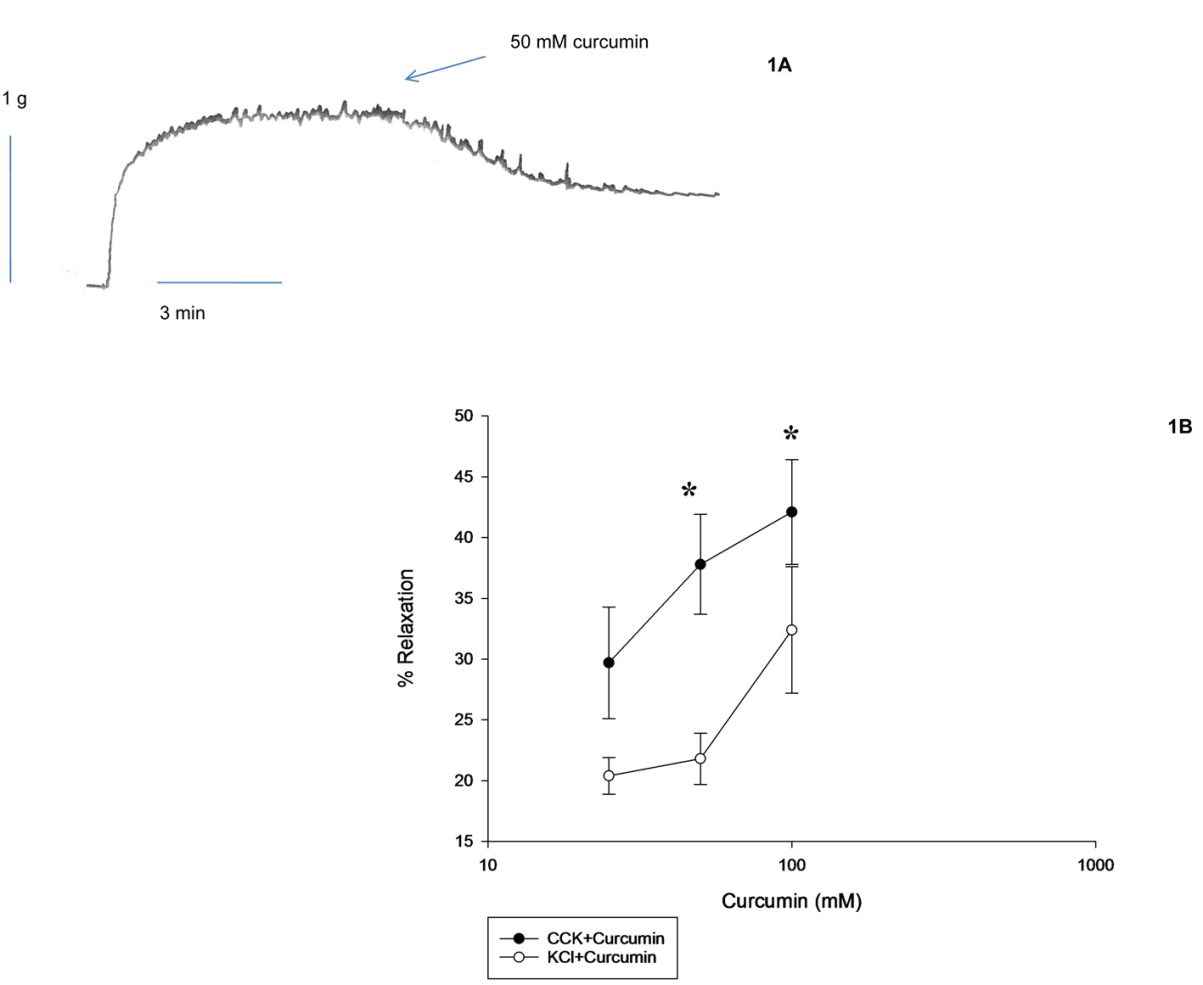
Effect of curcumin on CCK- or KCl-induced tension. (1A) A data trace showing the relaxation caused by curcumin on CCK-induced tension in a male guinea pig gallbladder strip. (1B) Curcumin (25 mM) was used to relax the CCK- or KCl-induced tension, there was no significant difference in the amount of curcumin-induced relaxation. When 50 mM curcumin was used there was a significant (P < 0.01, n = 8) difference in the amount of CCK-induced tension (filled circles) when compared to the amount of relaxation in the KCl-induced tension (open circles). When 100 mM curcumin was used, there was a significant (P < 0.05, n = 7) difference in the amount of curcumin-induced relaxation when CCK and KCl were compared. The CCK-induced tension was reduced more than the KCl-induced tension. There was no significant difference between the amount of CCK-induced tension and the KCl-induced tension. The significance was determined by paired *t*-tests.

Curcumin was found to have no cholecystokinetic effect in guinea pig gallbladder strips, i.e., none of the concentrations used (25, 50 or 100 mM) produced any tension in the gallbladder strips. CCK was used to generate tension and curcumin was added to the chambers to determine if curcumin induced a relaxation of the CCK-induced tension (Fig. 1A). Curcumin produced a concentration-dependent relaxation of CCK-induced tension (Fig. 1B). Curcumin also produced a concentration-dependent relaxation of KCl-induced tension (Fig. 1B). When 25 mM curcumin was used to relax either the CCK- or KCl-induced tension, there was no significant difference in the amount of relaxation observed (29.7 ± 4.6 vs. 21.3 ± 1.3%, n = 9). The amount of curcumin-induced relaxation using 50 mM curcumin was significantly (29.7 ± 4.6 vs. 21.8 ± 2.1%, n = 8, P < 0.001) greater when CCK was used rather than KCl. The amount of curcumin-induced relaxation using 100 mM curcumin was significantly (44.3 ± 4.0 vs. 27.0 ± 4.8%, n = 7, P < 0.01) greater in the CCK-induced tension than the KCl-induced tension (Fig. 1B). There was no significant difference in the tensions generated by CCK or KCl.

**Figure 2:**
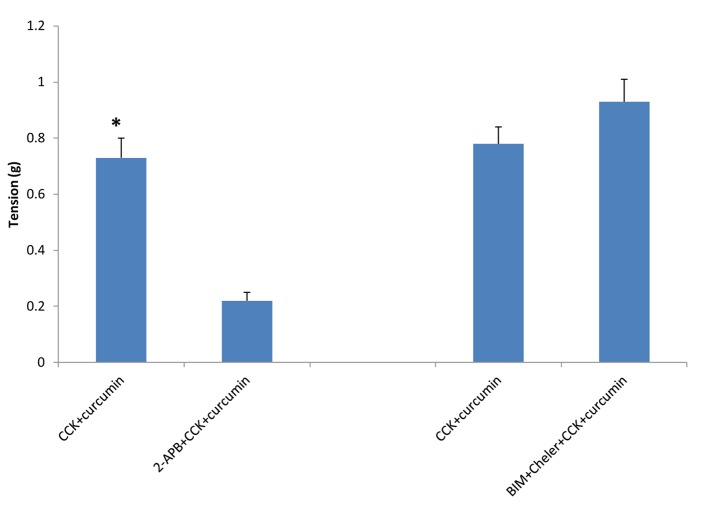
The effect of 2-APB or PKC blockers on curcumin-induced tension of CCK-induced tension. When the strips were treated with 2-APB, an inhibitor of IP_3_-induced Ca^2+^ release, there was a significant (P < 0.001, n = 4) decrease in the amount of CCK-induced tension. The use of the PKC blockers bisindolymaleimide IV and chelerythrine Cl^-^ had no significant effect on the amount of CCK-induced tension.

**Figure 3:**
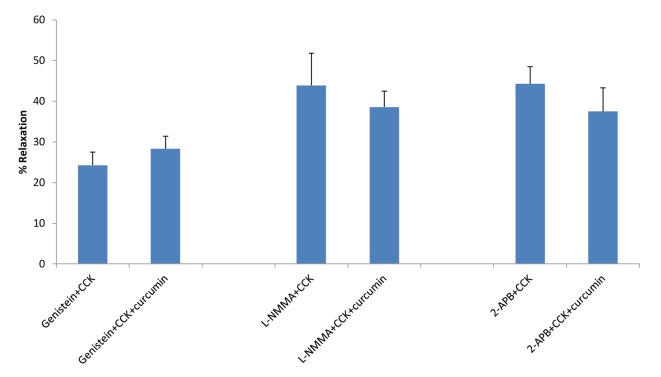
The effects of genistein, L-NMMA or 2-APB on curcumin-induced relaxation. Neither genistein, L-NMMA, nor 2-APB had a significant effect on the amount of curcumin-induced relaxation of CCK-induced tension.

The use of 2-APB, an inhibitor of IP_3_-induced Ca^2+^ release, caused a significant (P < 0.001) decrease in the amount of CCK-induced tension (0.73 ± 0.07 vs. 0.22 ± 0.03 g, n = 4, Fig. 2). The use of the PKC inhibitors BIM and chelerythrine Cl^-^ had no significant on the amount of CCK-induced tension with or without curcumin (Fig. 2). There was no significant change in the amount of relaxation observed between the strips not treated with 2-APB when compared to those treated with 2-APB (44.3 ± 4.2 vs. 37.5 ± 5.8%, n = 4, Fig. 3).

When the PKA inhibitor PKA-IM was used, there was a significant (P < 0.05) increase in the amount of CCK-induced tension (0.95 ± 0.05 vs. 1.01 ± 0.06 g, n = 4); however, there was no significant effect on the amount of curcumin-induced relaxation (38.1 ± 3.7 vs. 37.3 ± 4.1%, n = 4).

Treatment of the strips with KT5823, a PKG blocker, had no significant effect on the amount of CCK-induced tension (0.89 ± 0.08 vs. 0.83 ± 0.08 g, n = 4). KT5823 significantly (P < 0.001) increased the amount of curcumin-induced relaxation of CCK-induced tension (12.7±2.9 vs. 28.8±2.6%, n = 4). Genistein had no significant effect on the amount of curcumin-induced relaxation of CCK-induced tension (24.3±3.2 vs. 28.3±3.1%, n = 6, Fig. 3). There was a significant (P < 0.01) decrease in the amount of CCK-induced tension (0.76 ± 0.07 vs. 0.67 ± 0.06 g). The use of L-NMMA, a NO synthase blocker, significantly (P < 0.01) increased the amount of CCK-induced tension (0.81 ± 0.07 vs. 0.93 ± 0.06 g, n = 5), but had no significant effect on the amount of curcumin-induced relaxation (43.9±7.9 vs. 38.5±3.9%, n = 5, Fig. 3).

**Figure 4:**
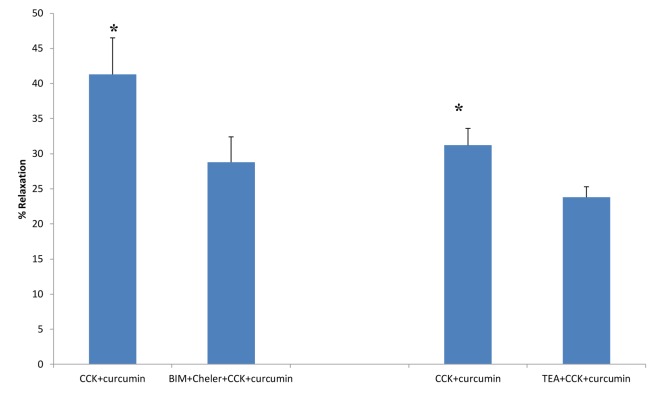
The effects of PKC blockers or TEA on curcumin-induced relaxation of CCK-induced tension. The use of the PKC inhibitors BIM (0.5 μM) and chelerythrine Cl^-^ (Cheler, 5.0 μM) significantly (P < 0.001; n = 5) decreased the amount of curcumin-induced relaxation. The use of TEA, a K^+^ channel blocker, significantly (P < 0.01, n = 5) decreased the amount of curcumin-induced relaxation of CCK-induced tension.

BIM and chelerythrine Cl^-^ are PKC inhibitors. The use of the PKC inhibitors caused a significant (P < 0.001; 41.3 ± 5.2 vs. 28.8 ± 3.6 g, n = 5; Fig. 4) decrease in the amount of curcumin-induced relaxation. However, there was no significant effect of the PKC inhibitors on the amount of CCK-induced tension (Fig. 2).

When TEA was added to the chambers prior to the CCK, a significant (P < 0.01; 31.2±2.4 vs. 23.8±1.5%, n = 5) decrease in the amount of curcumin-induced relaxation was observed (Fig. 4).

**Figure 5:**
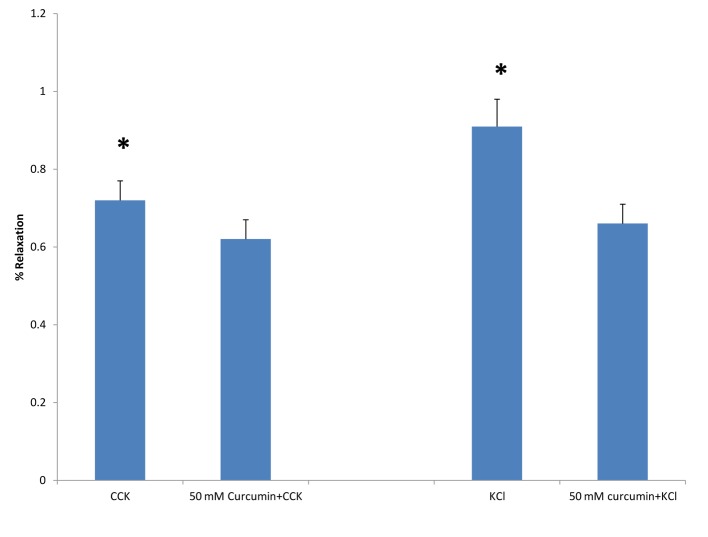
The effect of curcumin on CCK- or KCl-induced tension. When curcumin (50 mM) was added to the chambers 3 min prior to the addition of CCK (1 nM) there was a significant decrease (P < 0.01) in the amount of tension generated (n = 6). When 50 mM curcumin was added to the chambers 3 min prior to the addition of KCl (40 mM), there was a significant (P < 0.001) decrease in the amount of tension generated (n = 5).

When curcumin was added to the chambers 3 min prior to the addition of KCl there was a significant decrease (P < 0.001) in the amount of tension generated (0.84 ± 0.07 vs. 0.63 ± 0.04 g, n = 5; Fig. 5). When 50 mM curcumin was added to the chambers 3 min prior to the addition of CCK (1 nM), there was a significant (P < 0.01) decrease in the amount of tension generated (0.72 ± 0.05 vs. 0.62 ± 05 g, n = 6; Fig. 5).

## Discussion

In the present study the effects of curcumin on male guinea pig gallbladder motility were examined. The initial finding was that in the male guinea pig gallbladder strips curcumin had no stimulatory effect on the gallbladder strips. Instead a concentration-dependent relaxation of CCK- and KCl-induced tension was obtained using curcumin. In humans it was reported that 20 mg of curcumin swallowed with 100 mL of water was capable of contracting the human gallbladder (of both sexes) 29% within an observation time of 2 h [17, 18]. The concentration of curcumin used in the current study may not be considered physiologic; however, it has been argued that the short-term application of high concentrations of factors can mimic low (physiologic) levels applied over a long period of time.

Neither 2-APB nor PKA-IM had a significant effect on the amount of curcumin-induced relaxation; therefore, neither intracellular Ca^2+^ release nor PKA mediated the curcumin-induced relaxation. While the PKG blocker, KT5823, had no significant effect on the CCK-induced tension, it significantly increased the amount of curcumin-induced relaxation. This suggested that KT5823 was blocking on-going activity which allowed curcumin to have a greater effect.

Curcumin (10^-6^ - 10^-4^ M) has an anti-proliferative effect on vascular smooth muscle cells which was mediated through inhibition of protein tyrosine kinase. It also had apoptotic effects on the same cells which is mediated by PKC activity [6]. Curcumin suppressed the TPA-induced invasion of MCF-7 human breast cancer cells through inhibition of PKCα-dependent matrix metalloproteinase-9 [19, 20]. In the present study, genistein had no significant effect on the amount of curcumin-induced relaxation; therefore, protein tyrosine kinase does not mediate the curcumin-induced relaxation in guinea pig gallbladder strips.

Cheng et al [9] demonstrated that curcumin caused a concentration-dependent increase in the muscle tone in the urinary bladder of Wistar rats. The PKC blocker chelerythrine markedly reduced the curcumin-stimulated contraction of the rat urinary bladder. While no increase in tension was observed in the guinea pig gallbladder strips when curcumin was added to the chambers, the use of chelerythrine Cl^-^ and BIM significantly decreased the amount of curcumin-induced relaxation of CCK-induced tension. This suggested that PKC may mediate a part of the curcumin-induced relaxation of the gallbladder strips.

Curcumin has been shown to decrease intestinal motility in albino rats which may partially explain the traditional use of curcumin in disorders such as diarrhea, abdominal cramps and irritable bowel syndrome [21]. No mechanism of action was discussed. Curcumin has also been shown to decrease gastric emptying in albino rats. It was suggested that NO may mediate this effect; however, no evidence was presented [10]. Since L-NMMA had no significant effect on the amount of curcumin-induced relaxation, NO did not mediate the curcumin-induced relaxation in guinea pig gallbladder strips.

KCl was used to directly depolarize the gallbladder strips. Extracellular Ca^2+^ entry has been shown to be required to initiate CCK-induced tension [22, 23]. Nifedipine blocks L-type Ca^2+^ on smooth muscle [24]. Nifedipine virtually abolished spontaneous interdigestive gallbladder contractile activity and decreased resting gallbladder tone. This suggested that extracellular Ca^2+^ entry was important in gallbladder motility [22, 23, 25]. Curcumin, when added to the chambers prior to KCl, caused a significant decrease in the amount of KCl-induced tension. The blocking of L-type Ca^2+^ channels mediated part of the curcumin-induced relaxation. A similar significant decrease in CCK-induced tension was observed with curcumin. Since curcumin significantly decreased the amount of both KCl- and CCK-induced tension when added to the chambers prior to either agonist, it suggested that curcumin exerts its effect in part by blocking L-type Ca^2+^ channels.

Curcumin was reported to have an inhibitory effect on voltage-dependent K^+^ channels (K_v_) in rabbit arterial smooth muscle cells. Using the whole-cell patch clamp technique on freshly isolated coronary smooth muscle cells, Hong et al [5] demonstrated that curcumin inhibited K_v_ channels in a state-, time-, and use-dependent manner. In the present study the use of TEA significantly decreased the amount of curcumin-induced relaxation. The concentration used (5 μM) is within the range recommended by Vergara et al [26].

In conclusion, curcumin-induced relaxation is mediated by curcumin inhibiting L-type Ca^2+^ channels; thereby, probably activating other signaling pathways. In addition, K^+^ channel opening also mediated part of the curcumin-induced relaxation. Lastly, PKC also mediated a part of the curcumin-induced relaxation.
